# Molecular Detection of *Toxoplasma gondii* in Blood Samples of Domestic Livestock in the Republic of Korea

**DOI:** 10.3390/pathogens12040547

**Published:** 2023-04-01

**Authors:** Min-Jeong Ji, Hyung-Chul Cho, Yu-Jin Park, Dong-Hun Jang, Jinho Park, Kyoung-Seong Choi

**Affiliations:** 1 Department of Animal Science and Biotechnology, College of Ecology and Environmental Science, Kyungpook National University, Sangju 37224, Republic of Korea; 2 College of Veterinary Medicine, Jeonbuk National University, Iksan 54596, Republic of Korea

**Keywords:** *B1* gene, cattle, goats, nested PCR, *Toxoplasma gondii*

## Abstract

*Toxoplasma gondii*, a major zoonotic pathogen distributed worldwide, causes severe infections in humans, animals, and birds. However, limited information is available regarding *T. gondii* infection in livestock in the Republic of Korea (ROK). Herein, we determined the prevalence of *T. gondii* infection in livestock in the ROK and identified animal species that can potentially transmit *T. gondii* to humans. *B1* gene-targeting nested polymerase chain reaction detected *T. gondii* DNA in 3.3% (2/61), 2.9% (3/105), 14.1% (11/78), and 15.4% (14/91) of dairy cattle, beef cattle, Boer goats, and Korean native goats, respectively. The prevalence of *T. gondii* was significantly higher (*p* = 0.002) in goats than in cattle. The risk of contracting *T. gondii* infection was significantly higher by 6.18-fold in Korean native goats (95% confidence interval [CI]: 1.72–22.27%, *p* = 0.005) and by 5.58-fold in Boer goats (95% CI: 1.50–20.76%, *p* = 0.010) than in beef cattle. Our *T. gondii* DNA sequences exhibited 97.1–100% homology with those obtained from various hosts in other countries. To the best of our knowledge, this is the first study to report *T. gondii* infection using the blood samples of domestic ruminants in the ROK. The results revealed that the prevalence of *T. gondii* infection is higher in goats than in cattle as determined by molecular detection. Thus, these findings suggest that *T. gondii* can be transmitted from ruminants to humans via meat consumption.

## 1. Introduction

*Toxoplasma gondii* is an obligate intracellular parasite that can infect a wide range of host species [1]. According to the Food and Agriculture Organization of the United Nations, *T. gondii* constitutes the fourth leading cause of foodborne diseases and is a significant global public health concern [2]. Cats and wild felids comprise the only definitive hosts that excrete oocysts in their feces, whereas humans and other animals serve as intermediate hosts [3]. *Toxoplasma gondii* infection is primarily transmitted to intermediate hosts via the ingestion of tissue cysts from undercooked or raw meat, consumption of food and water contaminated with oocysts, or transplacental transmission [1,3,4,5].

The manifestations of *T. gondii* infection in humans range from asymptomatic conditions to chronic diseases, and include: abortion; severe congenital abnormalities, such as cerebral calcification, blindness, microcephaly, and seizure disorders; severe neuromuscular complications; pneumonia; and even death, in immunocompromised individuals and newborns [4,6,7,8]. Sheep and goats are more susceptible to *T. gondii* infection than cattle [9]. In small ruminants, *T. gondii* infection causes reproductive failures, such as stillbirths, abortion, fetal abnormalities, and postnatal mortality [8,10,11,12], consequently leading to economic losses in the livestock industry. Conversely, natural infection of cattle with *T. gondii* does not usually result in clinical symptoms or abortion [13]. However, cattle play important roles in the transmission of *T. gondii* infection to humans via the consumption of raw milk or of raw or undercooked meat, as these parasites leave the bloodstream and develop cysts in the muscle [13,14].

Serological tests and molecular-based methods have been primarily used to detect *T. gondii* infection. Serological assays include a modified agglutination test, indirect fluorescent antibody test, and enzyme-linked immunosorbent assay [15,16,17]. However, it is difficult to ascertain whether seropositivity indicates the presence of infective tissue cysts in various animal species and thus the risk of human infection. Conversely, among molecular-based techniques, polymerase chain reaction (PCR) targeting different genetic markers, including the glycerol-3-phosphate dehydrogenase (*B1*), internal transcribed spacer (*ITS-1)*, and 18S rDNA sequences, has been employed to detect *T. gondii* infection [18,19,20]. This method enables the early diagnosis of toxoplasmosis; however, the disadvantage is that it amplifies the DNA of both live and dead parasites [21].

*Toxoplasma gondii* has been classified into three genetic lineages based on its virulence: types I, II, and III [22]. The distribution of *T. gondii* varies with geographical region and host. Type I is more virulent in mice than types II and III [22,23,24,25]. Types II and III are more common in animal samples than type I, whereas type I and type I-like atypical isolates are more likely to cause severe retinochoroiditis [26] and disseminated toxoplasmosis, respectively, in immunocompetent patients [27].

Studies in other countries have been mainly conducted with serum samples of small ruminants and the prevalence of *T. gondii* has been reported to be relatively high among the ovine population [3,6,28,29,30,31,32]. However, compared with other Asian countries, scarce information is available regarding *T. gondii* infection in livestock in the Republic of Korea (ROK). Considering that meat-producing animals constitute an important source of human infection, there is an urgent need to monitor the infection rate of *T. gondii* in various livestock species. Therefore, this study aimed to determine the prevalence of *T. gondii* in the blood of livestock in the ROK using the *B1* gene and to identify the animal species that can potentially transmit this infection to humans.

## 2. Materials and Methods

### 2.1. Blood Collection

**Figure 1:**
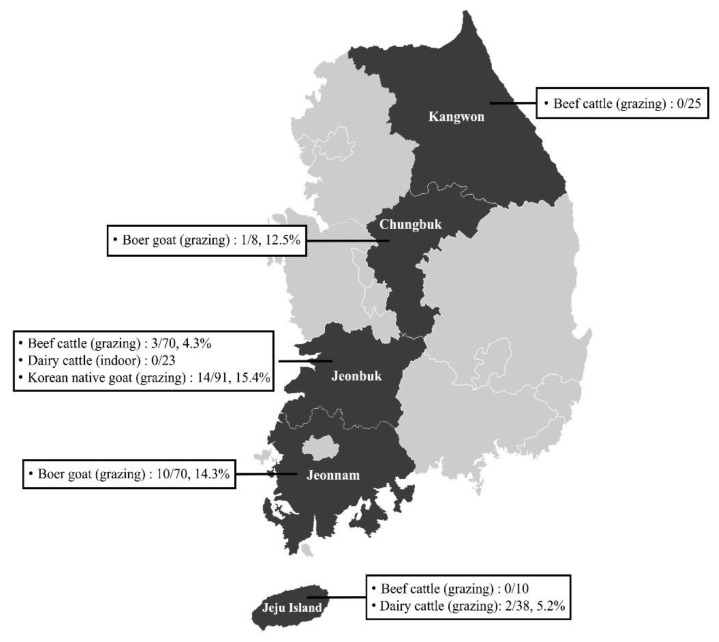
Map of the farms selected for sample collection in the Republic of Korea.

From October 2014 to June 2017, 335 blood samples were collected from healthy dairy cattle (*n* = 61), beef cattle (*n* = 105), Korean native goats (*n* = 91), and Boer goats (*n* = 78) on randomly selected farms in five different regions (Kangwon, Chungbuk, Jeonbuk, and Jeonnam provinces and Jeju Island) of the ROK (Figure 1). Each blood sample was collected in a collection tube with anticoagulant (BD Vacutainer^®^, Franklin Lakes, NJ, USA) and immediately delivered to the laboratory.

### 2.2. DNA Extraction, Genomic Detection, and Sequencing

DNA was extracted from 200 μL of each blood sample using DNeasy Blood Kit (Qiagen, Valencia, CA, USA) according to the manufacturer’s instructions. The extracted DNA was stored at −80 °C until use. *Toxoplasma gondii* was screened via nested PCR targeting the highly conserved *B1* gene under the following conditions: 94 °C for 5 min; followed by 35 cycles of denaturation at 94 °C for 1 min, annealing at 52 °C for 1 min, and extension at 72 °C for 1 min; and a final extension step at 72 °C for 10 min [33]. *Toxoplasma gondii* DNA was provided by Dr. Young-Ha Lee (School of Medicine, Chungnam National University) and was used as positive DNA when the *T. gondii* DNA was amplified from the samples. For all the reactions, negative and positive controls were included. The amplified PCR products (530 bp) were separated via electrophoresis on a 1.5% agarose gel and visualized after staining with ethidium bromide.

### 2.3. Sequencing and Phylogenetic Tree

All secondary PCR products were purified using AccuPower PCR Purification Kit (Bioneer, Daejeon, ROK) and used for direct sequencing (Macrogen, Daejeon, ROK). PCR amplicons were sequenced using BigDye Terminator 3.1 Cycle Sequencing Kit on a 3500 xL Genetic Analyzer (Applied Biosystems, Foster City, CA, USA) according to the manufacturer’s instructions with the same primer set that was used for conventional PCR. The nucleotide sequences obtained in this study were aligned with those in the GenBank database using ClustalX (v.2.0) and compared with global references for *T. gondii*. Phylogenetic analysis was performed via the maximum-likelihood method implemented in MEGA11 using the best substitution model. Bootstrap values were calculated by analyzing 1000 replicates to evaluate the reliability of the clusters. The model used in this study was Jukes Cantor (JC) + G + I. The nucleotide sequences obtained herein were deposited in the GenBank database under the accession numbers ON648716−ON648733.

### 2.4. Statistical Analysis

Exact confidence intervals (CIs) for prevalence at the 95% level were calculated. Data were analyzed using SPSS Statistics 25 software package for Windows (SPSS Inc., Chicago, IL, USA). Chi-square test (χ^2^) was used to determine any association between the prevalence of *T. gondii* and risk of infection in animal species. Univariate binary logistic regression analysis was performed to evaluate the association of *T. gondii* infection and species. The odds ratio and 95% CI were calculated to determine the probability of association detection. A *p*-value of ≤0.05 was considered statistically significant.

## 3. Results

### 3.1. Prevalence of T. gondii in Ruminants

Among the 335 ruminant blood samples examined, 30 were positive for *T. gondii*, with an overall prevalence of 9.0% (95% CI: 5.9–12.0%), based on PCR analysis targeting the *B1* gene. The prevalence of *T. gondii* was the highest in Korean native goats (15.4%) and the lowest in beef cattle (2.9%; Table 1). Furthermore, the prevalence of *T. gondii* was significantly higher (*p* = 0.002; Table 1) in goats (14.8%, 25/169) than in cattle (3.0%, 5/166). The risk factor associated with *T. gondii* infection in animal species was determined using univariate binary logistic regression analysis (Table 1). Korean native goats and Boer goats were at higher risk of *T. gondii* infection than cattle. The risk of contracting *T. gondii* infection was significantly higher by 6.18-fold in Korean native goats (95% CI: 1.72–22.27%, *p* = 0.005) and by 5.58-fold in Boer goats (95% CI: 1.50–20.76%, *p* = 0.010) than in beef cattle. Statistical analysis revealed that dairy cattle were not at higher risk of *T. gondii* infection than beef cattle.

### 3.2. Sequence and Phylogenetic Analysis

**Figure 2:**
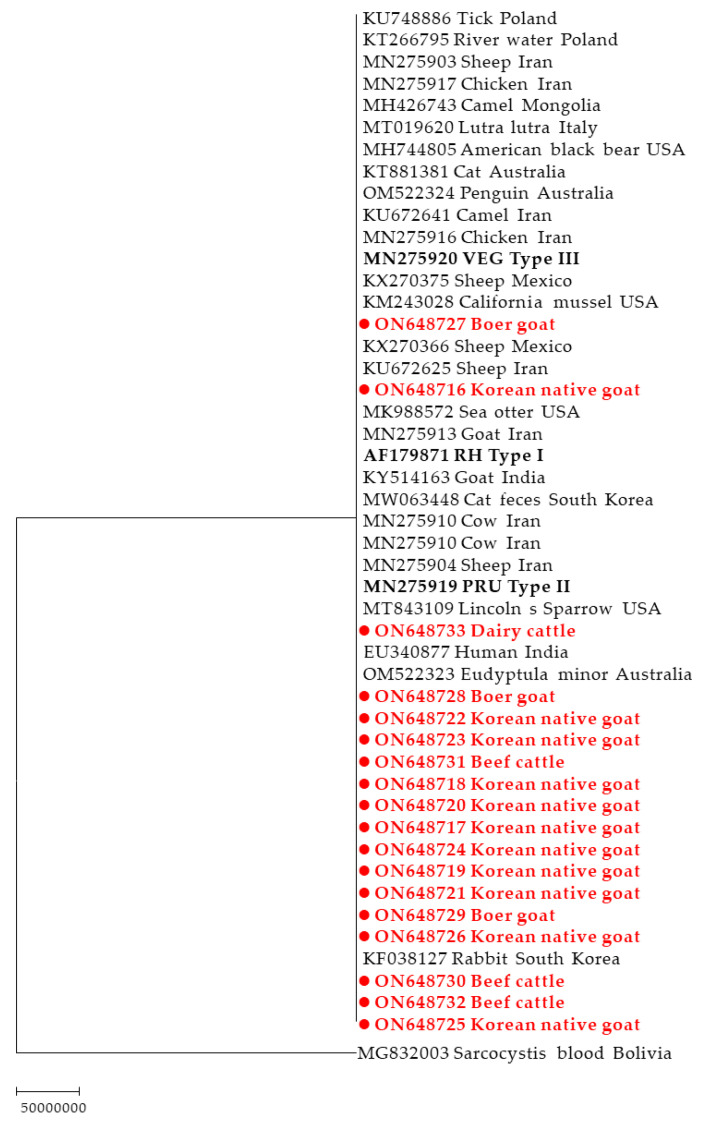
Phylogenetic tree based on the *B1* gene of *T. gondii* constructed via the maximum-likelihood method using the JC + G + I model. The numbers over the branches indicate bootstrap values as a percentage of 1000 replicates that support each phylogenetic branch. Only bootstrap values exceeding 70% are presented. The sequences marked with circles and red boldface type were determined in this study.

From the 30 positive samples, 18 good amplicons were obtained. The amplicons sequenced from beef cattle, dairy cattle, Boer goats, and Korean native goats were 3 (100%), 1 (50%), 3 (27.3%), and 11 (78.6%), respectively (Table 1). The *T. gondii* sequences in this study exhibited 96.5–99.8% homology with each other (Table S1). The 18 sequences were compared with those from various hosts reported in other countries, including sheep, goats, cows, humans, ticks, cats, birds, water, and mussels, showing a relatively high nucleotide sequence homology of 97.1–100% (Table S1). The sequences obtained in this study exhibited 97.6–99.8% and 97.4–99.4% homology with those obtained from cat feces and rabbits, respectively, which were previously reported in Korea (Table S1). Phylogenetic analysis revealed that these 18 sequences formed one cluster with other *T. gondii* strains (Figure 2), exhibiting no discrimination according to types. The sequences of the reference strains RH (type I), PRU (type II), and VEG (type III) were extremely similar, with differences in only two nucleotides (data not shown).

## 4. Discussion

To the best of our knowledge, this is the first study to detect *T. gondii* infection in blood samples collected from domestic livestock in the ROK. Based on our findings, the infection rate of *T. gondii* was higher in goats (14.8%) than in cattle (3.0%). Our prevalence results in goats were consistent with those reported in Iran (11.7%), Israel (11.6%), and Algeria (18.68%) [16,29,34], whereas the prevalence of *T. gondii* in cattle in this study was relatively lower than that reported in Egypt (13.46%), Iran (56%), Israel (7.5%) Pakistan (12.2%), Poland (10.2%), and Tunisia (19.3%) [4,6,13,29,35,36]. The reason for this difference in cattle remains unclear. However, it could be due to the difference in the sample sources used to detect *T. gondii*. Although blood is generally used for serological analysis, other studies frequently use tissue samples, such as those of the brain, diaphragm, heart, liver, muscle, and tongue for *T. gondii* detection in cattle [4,29,35,36]. A previous study reported that *T. gondii* diagnosis using blood samples can yield different results, explaining that infection of host with tachyzoites, bradyzoites, and sporozoites considerably affects the time of blood infection and its persistence [37]. Moreover, the presence of the parasite at the tachyzoite stage represents acute phase infection [37]. Considering that the vast majority of *T. gondii* infections in numerous host animals, including the animals investigated in this study, were asymptomatic, these animals were considered a potential source of *T. gondii* infection to humans. Therefore, these results highlight the importance of the early detection of *T. gondii* and the need for a surveillance program to prevent human infections.

In this study, the prevalence of *T. gondii* in cattle in the ROK was lower than that reported in Egypt (13.5%) [6], Iran (56%) [4], and Pakistan (12.2%) [13] based on molecular analysis. The low prevalence may be attributed to different geographical locations and management systems (breeding, feeding, and water supply source) for animal production. The cattle used in this study were primarily grazing in a pasture. Previous studies have reported that grazing increases the risk of exposure to *T. gondii* in cattle [38,39]. Pasture contaminated with oocysts due to cats constitutes the primary transmission route of *T. gondii* for cattle [38]. However, our findings were inconsistent with those reported in previous studies [38,39]. At this point, we cannot explain the exact reason for this low prevalence. It is speculated that cats would not have had much access to the pastures, thereby leading to less oocyst contamination of the pastures. Moreover, according to our results, there was no significant difference in the prevalence of *T. gondii* between beef and dairy cattle (Table 1). Although no precise conclusions can be drawn due to the limited number of samples, the breeding system in the current study did not appear to significantly affect *T. gondii* infection. Furthermore, it is uncertain whether the prevalence of *T. gondii* in cattle was low only in the areas examined or if the infection rate was generally low in the ROK. Therefore, large-scale epidemiological studies are warranted to determine the association between the prevalence of *T. gondii* and management or cattle breeding systems.

In the ROK, goat meat is considered a health supplement, and thus, the number of goats raised has been increasing [40]. Small ruminants are intermediate hosts of *T. gondii* and are highly susceptible [41]; even adult goats die of toxoplasmosis [42]. Unlike cattle, goats often graze on grass close to the soil and feed on shrub leaves [34], which increase their vulnerability to *T. gondii* infection. The high prevalence of *T. gondii* in goats reported herein is closely related to management systems (contact with other animals or contamination of soil). Thus, this raises the possibility that there are more cats in goat farms than in cattle farms. The presence of cats in the barn increases the risk of exposure to *T. gondii* through the dispersal of millions of oocysts into the environment, especially soil; this results in oocysts contaminating the soil, which increases *T. gondii* infection [43,44]. *Toxoplasma gondii* is a major pathogen that causes abortion and neonatal mortality in goats [45], leading to significant economic loss. Nevertheless, its importance has been neglected in the ROK. Consequently, our results suggest that goats may serve as a major source of *T. gondii* transmission to humans owing to the steady increase in meat consumption. Therefore, it is necessary to understand the significance of this infection in goats, its impact on public health, and its control.

In this study, we detected *T. gondii* infection by amplifying and sequencing the *B1* gene. The *B1* is a multicopy, highly conserved gene and is used for detecting *T. gondii* DNA from various samples [26,46]. The advantage of this gene is its high specificity and sensitivity, facilitating the detection of 50 fg *T. gondii* DNA [20,26]. Although the number of parasites was not estimated in this study as qPCR was not performed, the results are indicative of the infection status in animals, suggesting that the PCR procedure targeting the *B1* gene is suitable for the routine detection of *T. gondii*. In addition, compared with the serological test results, the PCR results indicate active infection, which can help establish effective control strategies for *T. gondii*.

Currently, to the best of our knowledge, there is no information available for the genetic characterization of *T. gondii* in livestock in the ROK. Based on the phylogenetic analysis of the *B1* gene, the sequences detected in this study belonged to the same group, with samples including types I and III. A limitation of this study is that a more accurate genotype of *T. gondii* could not be revealed because genotype analysis was not performed. Further studies are warranted to identify the sources (cat, rodents, food, water, or wildlife access to farms) of *T. gondii* infection, specific genotypes of *T. gondii* circulating in the ROK, and association between pathogenicity and genotype.

## 5. Conclusions

This study revealed the genomic detection of *T. gondii* in domestic ruminants in the ROK. Our findings demonstrated that the prevalence of *T. gondii* infection is higher in goats than in cattle, indicating that *T. gondii* infection may be affected by the rearing system of the animals. The detection of *T. gondii* in the blood suggests that the meat of domestic ruminants is a potential source of *T. gondii* infection transmission to humans. The results of this study will aid in establishing a strategy to control and manage *T. gondii* infection.

## Figures and Tables

**Table 1:** Prevalence of *T. gondii* infection among ruminants.

Species (No. of Samples Sequenced)	No. of *T. gondii* Positive Samples	χ^2^ (*p-*Value)	OR	95% CI	*p*-Value
Beef cattle (3)	3/105 (2.9%)	14.348 (0.002)	1.000	-	-
Dairy cattle (1)	2/61 (3.3%)		1.153	0.187–7.097	0.878
Boer goats (3)	11/78 (14.1%)		5.582	1.501–20.756	0.005
Korean native goats (11)	14/91 (15.4%)		6.182	1.716–22.269	0.010

OR: odds ratio, CI: confidence interval.

## Data Availability

The datasets generated during the current study are available in the GenBank repository at Banklt ID 2588636 [https://https://www.ncbi.nlm.nih.gov/WebSub/?form=history&tool=genbank, accessed on 22 February 2023], accession numbers ON648716–ON648733. These accession numbers will be released after 6 months.

## Supplementary Materials

The following supporting information can be downloaded at: https://www.mdpi.com/article/10.3390/pathogens12040547/s1, Table S1: Comparison of nucleotide sequence homology between *T. gondii* from other animals and the sequences obtained in this study.

## Abbreviations

CI	confidence interval
PCR	polymerase chain reaction
ROK	Republic of Korea
*T. gondii*	*Toxoplasma gondii*
